# Exploring clonality and virulence gene associations in bloodstream infections using whole-genome sequencing and clinical data

**DOI:** 10.3389/fcimb.2023.1274573

**Published:** 2023-11-14

**Authors:** Claudio Neidhöfer, Marcel Neuenhoff, Robert Jozič, Brenda Atangcho, Sandra Unsleber, Ulrike Neder, Silke Grumaz, Marijo Parčina

**Affiliations:** ^1^ Institute of Medical Microbiology, Immunology and Parasitology. University Hospital Bonn, Bonn, Germany; ^2^ Institute of Experimental Haematology and Transfusion Medicine, University of Bonn, Bonn, Germany; ^3^ Bioinformatics and Systems Biology, Justus Liebig University Giessen, Giessen, Germany; ^4^ Institute for Functional Gene Analytics, Bonn-Rhein-Sieg University of Applied Sciences, Sankt Augustin, Germany; ^5^ Noscendo GmbH, Duisburg, Germany

**Keywords:** bloodstream infections, whole-genome sequencing, clonality patterns, virulence factors, resistance genes, genotype-phenotype correlation, genomic characterization of BSI, molecular epidemiology of BSI

## Abstract

**Background:**

Bloodstream infections (BSIs) remain a significant cause of mortality worldwide. Causative pathogens are routinely identified and susceptibility tested but only very rarely investigated for their resistance genes, virulence factors, and clonality. Our aim was to gain insight into the clonality patterns of different species causing BSI and the clinical relevance of distinct virulence genes.

**Methods:**

For this study, we whole-genome-sequenced over 400 randomly selected important pathogens isolated from blood cultures in our diagnostic department between 2016 and 2021. Genomic data on virulence factors, resistance genes, and clonality were cross-linked with *in-vitro* data and demographic and clinical information.

**Results:**

The investigation yielded extensive and informative data on the distribution of genes implicated in BSI as well as on the clonality of isolates across various species.

**Conclusion:**

Associations between survival outcomes and the presence of specific genes must be interpreted with caution, and conducting replication studies with larger sample sizes for each species appears mandatory. Likewise, a deeper knowledge of virulence and host factors will aid in the interpretation of results and might lead to more targeted therapeutic and preventive measures. Monitoring transmission dynamics more efficiently holds promise to serve as a valuable tool in preventing in particular BSI caused by nosocomial pathogens.

## Introduction

Bloodstream infections (BSIs) pose a substantial global health threat, leading to increased morbidity and mortality rates (McNamara et al., 2018). While identifying the causative pathogen and its antibiotic susceptibility remains a clinical priority, exploring additional factors such as genetic relatedness, virulence genes, and antibiotic resistance genes can significantly enhance patient outcomes and alleviate the overall burden (Wren, 2000; Leavis et al., 2006; Wyres et al., 2020; Allen et al., 2021). Whole-genome sequencing (WGS) studies have revolutionized our understanding of pathogen identification, antibiotic resistance, and epidemiology (Wren, 2000). Insights into clonality patterns can guide interventions to curtail the dissemination of specific strains and inform targeted prevention strategies (Wren, 2000; Leavis et al., 2006; Wyres et al., 2020). Furthermore, studying pathogen clonality provides valuable insights into the evolutionary dynamics of BSI pathogens, facilitating the prediction of future trends in antibiotic resistance and virulence (Wren, 2000; Allen et al., 2021).

Virulence factors play a crucial role in the colonization, invasion, and evasion of the host immune system by pathogens. Understanding the specific virulence factors associated with BSI-causing bacteria provides insights into disease mechanisms, severity, and potential therapeutic targets (Wren, 2000; L Thomas and Lee, 2012; Wyres et al., 2020). In-depth investigations of bacterial virulence factors associated with BSI have yielded significant findings concerning pathogenesis and host–pathogen interactions (Wren, 2000; Leavis et al., 2006; L Thomas and Lee, 2012; Wyres et al., 2020). Studying resistance genes and correlating them with phenotypical susceptibility are of paramount importance for addressing key research questions in the field of antimicrobial resistance leading to a comprehensive understanding of the interplay between genotype and phenotype (Wren, 2000; L Thomas and Lee, 2012; Mahfouz et al., 2020; Allen et al., 2021). It enables the validation and verification of resistance mechanisms and provides a more accurate assessment of the clinical implications of specific genetic variants.

In this study, we conducted a comprehensive whole-genome sequencing of over 400 randomly selected common BSI-causing pathogens to elucidate clonality patterns and assess the clinical significance of virulence and resistance genes, including their association with mortality. Our study findings advance the understanding of BSI pathogenesis and hold implications for more targeted therapeutic interventions.

## Methods

### Study design and data collection

For this study, over 400 bacterial isolates of common pathogens detected in blood cultures between January 2019 and December 2021 in our microbiological diagnostic unit, which services a tertiary referral and maximum care hospital and other hospitals in the area, were randomly selected for the genera *Acinetobacter*, *Bacteroides*, *Citrobacter*, *Enterobacter*, and *Serratia* and the species *Enterococcus faecalis*, *Enterococcus faecium*, *Escherichia coli*, *Klebsiella pneumonia*, *Proteus mirabilis*, *Pseudomonas aeruginosa*, *Staphylococcus aureus*, *Stenotrophomonas maltophilia*, *Streptococcus pneumoniae*, and *Streptococcus pyogenes* from cryo-storage. These were thawed, subcultured twice on Columbia 5% sheep blood agar (Becton Dickinson, Heidelberg, Germany), and inspected by two experienced operators prior to sequencing. Isolate information was complemented by accessible laboratory information on phenotypical susceptibility, growth of additional bacteria in the same blood culture, and routine diagnostic resistance gene detection, as well as accessible patient information on age, sex, hospitalization, 30-day mortality, and outcome. We constructed a database that was password-protected and accessible by only three operators who ensured that all patient data were fully de-identified prior to analysis. Unless specifically mentioned or reported, the minimum inhibitory concentrations (MICs) were determined by the VITEK 2 system (bioMérieux, Marcy-l’Etoile, France). Isolate susceptibility was inferred based on EUCAST Cl. Br. Tables v. 13.0.

### DNA preparation and sequencing

DNA isolation, library preparation, sequencing, and sequence assembly were carried out by Noscendo GmbH, Germany. Genomic DNA was prepared from pellets obtained from 5 ml of culture in a brain heart infusion (BHI) medium (Becton Dickinson, Heidelberg, Germany). Cell pellets were prepared, shipped on dry ice, and stored at −80°C until further processing. DNA was isolated with the ZymoBIOMICS DNA Miniprep Kit (Zymo Research, Irvine, CA, USA) according to the manufacturer’s instructions with a Vortex Genie 2 device equipped with an SI-H524 horizontal tube holder (Scientific Industries, Bohemia, NY, USA) to perform mechanical cell disruption for 10 min. DNA concentration was measured with the Qubit 1X dsDNA Assay-Kit on a Qubit 2.0 instrument (Thermo Fisher Scientific, Waltham, MA, USA), and size distribution was checked with the Agilent Genomic DNA 50 kb Kit on a 5200 Fragment Analyzer System (Agilent Technologies, Santa Clara, CA, USA). The libraries were prepared using the Ligation Sequencing Kit SQK-LSK109 with Native Barcoding Expansion 1–12 (PCR-free) EXP-NBD104 and Native Barcoding Expansion 13–24 (PCR-free) EXP-NBD114 according to the manufacturer’s instructions (Oxford Nanopore Technologies, Oxford, UK) together with NEB Blunt/TA Ligase Master Mix and NEBNext Companion Module for Oxford Nanopore Technologies Ligation Sequencing (New England Biolabs, MA, USA). Then, the libraries were prepared, pooled in equimolar ratio, quality checked, loaded on a MinION Flow Cell (R9.4.1), and finally sequenced on a MinION benchtop sequencer (Mk1B). For each batch preparation, a pellet of a 1.5-ml overnight culture (BHI medium) of DSM 1576–*Escherichia coli* was used as an internal quality control during the whole process, starting with DNA isolation.

### Bioinformatics analysis

After sequencing, fast5 data were basecalled using the Oxford Nanopore Technologies neural-network-based basecalling software Guppy (version 5.0.7) applying a high accuracy mode (config dna_r9.4.1_450bps_hac.cfg). Fastq statistics on read length distributions, expected coverages, and N50 values were calculated. Following closely the manual of the tool Trycycler (Wick et al., 2021) (https://github.com/rrwick/Trycycler, version 0.4.2), long-read consensus assemblies were produced from multiple input assemblies of the same input data set by using the following assemblers: Flye (https://github.com/fenderglass/Flye, version 2.8.3), Miniasm and Minipolish (https://github.com/rrwick/Minipolish, version 0.1.3), and Raven (https://github.com/lbcb-sci/raven, version 1.4.0). Before assembly, reads below a length of 1,000 bp were removed using the tool Filtlong (https://github.com/rrwick/Filtlong, version 0.2.0), at most removing 5% of the original data set. Nine subsamples were created, providing a minimum read depth of 100×. In cases in which average coverages were below 100×, the minimum read depth after subsampling was lowered to 50×. Subsampled data sets were then forwarded to the assemblers, producing nine assemblies in total, three of every assembler. The Trycycler then clustered similar contigs to detect spurious, incomplete, or misassembled contigs. After manual inspection, the conspicuous clusters were removed. The remaining clusters were further processed to result in circular chromosomes and plasmids. Consensus sequences were polished using Medaka (https://github.com/nanoporetech/medaka, version 1.4.3) to increase base accuracy and minimize assembly and sequencing errors.

Assembly quality was finally evaluated by Busco (https://busco.ezlab.org/, https://gitlab.com/ezlab/busco/-/releases/5.2.1, version 5.2.1). Furthermore, reads were mapped to the consensus sequence by using Minimap2 (https://github.com/lh3/minimap2, version 2.18) and Flye-samtools to get coverage values of every consensus base. Assemblies having a Busco completeness value above 95% and every consensus base covered with at least 5× coverage were regarded as high quality. All isolates with valid results and sufficient coverage were included in the analysis.

Detection of antimicrobial resistance (AMR) genes, stress response genes, and virulence factors was detected with AMRFinderPlus (3.11.4) “–plus” option/NCBI reference gene database (2023-02-23.1) (Feldgarden et al., 2021; Feldgarden et al., 2022). If species were present in the list of curated organisms, the “–organism” option was used to ignore universal species mutations and resistance genes and to screen for known point mutations. Only results with identity and coverage >90% were included. For further analysis, assemblies were imported into bactopia (Shen, ; Ewels et al., 2020; Petit and Read, 2020). FastANI (v1.33) was used to calculate the average nucleotide identity within species (Jain et al., 2018). MLST types were determined with the PubMLST database using MLST (2.23.0, https://github.com/tseemann/mlst) (Jolley et al., 2018). For *Enterobacterales*, plasmids were detected using plasmidFinder (2.1.6) (Camacho et al., 2009; Carattoli et al., 2014).

Strain-specific tools were used to obtain more in-depth information: *E. coli*—ECTyper (1.0.0) (Iguchi et al., 2015), *K. pneumoniae*—Kleborate (2.3.2) (Lam et al., 2021), *P. aeruginosa*—pasty (1.0.2, https://github.com/rpetit3/pasty) (Camacho et al., 2009; Thrane et al., 2016), and *S. pneumoniae*—Seroba (1.0.2) (Epping et al., 2018).

Phylogenetic trees and ANI values in **supplementary data** were created with Anvi’o v7.1 (Eren et al., 2021) using “anvi’o pangenomics workflow” (Edgar, 2004; Hyatt et al., 2010; Buchfink et al., 2015; Pritchard et al., 2016; Chan and Lowe, 2019). All detected resistance genes, stress response genes, and virulence factors for each species can be found in the **Supplementary Material.** (Sequence names are mentioned yellow for findings with shared gene symbol but multiple possible alleles. These were merged for our purposes if the genes belong to the same subclass.)

All data relevant to the study are included in the article or uploaded as **supplementary information.** Due to collaboration agreements, genomes have not been uploaded to any publicly accessible platform but can be shared upon reasonable request.

The Ethics Committee of the University Hospital Bonn confirmed that no ethics approval was required for this study.

## Results

### Isolate and patient information

The 364 isolates for which genomes were available with satisfactory coverage and quality belonged to 364 different patients of which the majority were men (60.16%). Detailed information on patients and isolates is listed in **Table 1**. In 55 cases (15.11%), the pathogen under study was not the only one isolated from the respective blood culture. In 7, there were 2 additional pathogens, and in another three, 3. Fourteen isolates were methicillin-resistant *S. aureus* (MRSA), 10 were vancomycin-resistant *enterococci* (VRE), and 26 were gram-negative rods falling into the German guideline classification of multidrug-resistant gram-negative rods on the basis of resistance against three (3MRGN) or four (4MRGN) of the following antibiotic groups: acylureidopenicillins, third- and fourth-generation cephalosporins, carbapenems, and fluoroquinolones.

**Table 1:** Patient and isolate information.

Age (years) Mean (min, max)	62.15 (0, 95)	
Sex		
**Female** **Male**	145 219	39.84% 60.16%
Ward type		
**Emergency center** **ICU** **Non-ICU ward** **Outpatient clinics**	92 112 154 6	25.28% 30.77% 42.31% 1.65%
Clinic		
**Anesthesiology** **Emergency departments** **General surgery** **Rehabilitation** **Gynecology** **Heart surgery** **Internal medicine** **Neonatology** **Neurosurgery** **Neurology** **Oncology** **Orthopedics** **Pediatrics** **Urology** **Others**	20 89 19 49 8 10 77 6 8 17 40 5 10 4 2	5.49% 24.45% 5.22% 13.46% 2.2% 2.75% 21.15% 1.65% 2.2% 4.67% 10.99% 1.37% 2.75% 1.1% 0.55%
Year		
**2016** **2017** **2018** **2019** **2020** **2021**	77 71 83 62 61 10	21.15% 19.51% 22.80% 17.03% 16.76% 2.75%
Isolates		
***Acinetobacter* spp.** ***Bacteroides* spp.** ***Citrobacter* spp.** ***Enterobacter* spp.** ***Enterococcus faecalis*** ***Enterococcus faecium*** ***Escherichia coli*** ***Klebsiella pneumoniae*** ***Proteus mirabilis*** ***Pseudomonas aeruginosa*** ***Serratia* spp.** ***Staphylococcus aureus*** ***Stenotrophomonas maltophilia*** ***Streptococcus pneumoniae*** ***Streptococcus pyogenes***	12 4 15 45 19 19 40 40 20 38 27 48 5 13 18	3.3% 1.1% 4.12% 12.36% 5.22% 5.22% 10.99% 10.99% 5.5% 10.44% 7.42% 13.19% 1.37% 3.57% 4.95%

### Species characteristics

**Figure 1:**
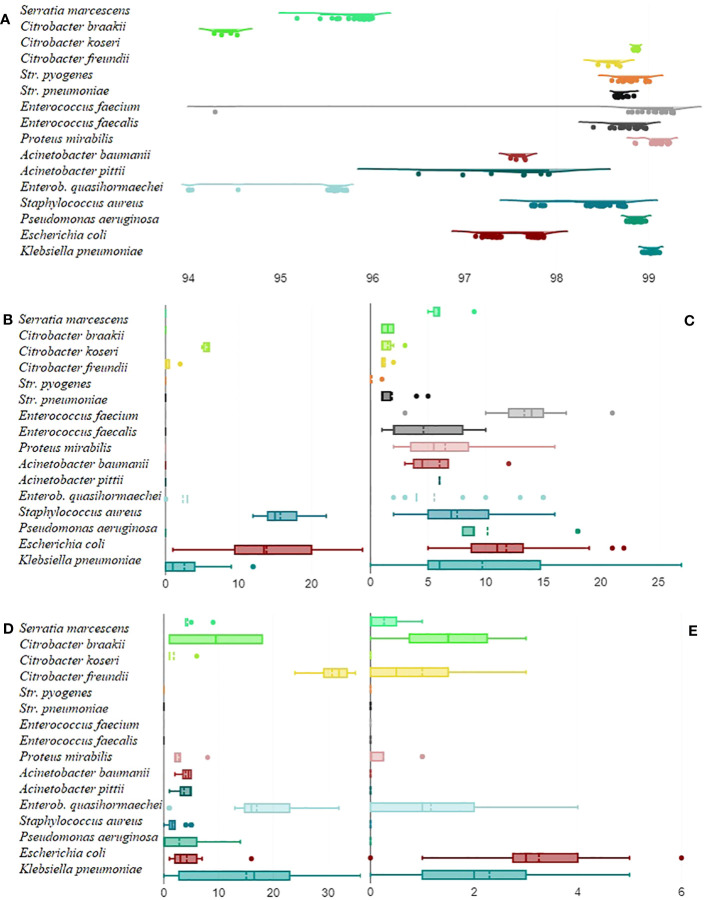
Mean average nucleotide identity by pathogen. For each isolate, the average nucleotide identity it had to all the other isolates within the same group in percent (*x*-axis) was calculated and displayed as a raincloud plot **(A)**. Boxplots on the number (*x*-axis) of virulence genes **(B)**, AMR genes **(C)**, stress response genes **(D)**, and plasmids **(E)** that pathogens carried.

The most frequently detected AMR genes were *fosA* (107), *oqxA* (73), and *emrD* (70). These were, however, only detected in *Enterobacterales* and *P. aeruginosa* that made up a large part of all isolates. An overview of detected virulence, stress response, and AMR genes can be found in the **Supplementary Material.** **Figure 1** displays to what degree the average nucleotide identity could vary across different pathogens and the number of virulence genes, AMR genes, stress response genes, and plasmids that different pathogens carried. The 30-day and 90-day mortality did not correlate with the number of resistance, AMR, or stress response genes but with patient age (rpb = 0.22, *n* = 248, *p* = 0.001 and rpb = 0.25, *n* = 201, *p* = <0.001, respectively).

#### Enterococcus faecalis

The majority of the isolates originated from patients in intensive care units (ICUs) with eight cases, followed by the emergency center (EC) with four cases. None of the isolates were identified as VRE either phenotypically or genotypically. There were 11 unique multilocus sequence typing (MLST) types represented once and four types represented twice. The isolates showed an average nucleotide identity of 98.78% to each other, ranging from 98.40% to 98.97%. Notably, patients infected with *E. faecalis* isolates encoding *dfrF*, *gyrA_S83I*, and *parC_S80I* exhibited significantly lower 30-day (all *p* = 0.015) and 90-day survival rates (all *p* = 0.047), although the assumptions for the *χ* ^2^ test were not met due to low cell frequencies. Out of all the isolates, only two showed higher MICs for trimethoprim/sulfamethoxazole than the threshold of ≤10, and both of these isolates tested positive for the *dfrG* gene.

#### Enterococcus faecium

Out of the *E. faecium* isolates, 10 were classified as VRE, but their presence was not associated with a lower 30-day or 90-day survival rate. Among these isolates, the MLST types of seven could not be determined, while six belonged to ST117 and three to ST80. Five ST117 isolates and two ST80 isolates were identified as VRE. No significant differences were observed in the 30-day and 90-day survival rates based on the presence or absence of specific genes. However, a positive correlation was found between survival and younger age (rpb = 0.71, *p* = 0.001). The isolates displayed an average nucleotide identity of 98.84% to each other, with a range of 94.29% to 99.27%.

#### Staphylococcus aureus

Fourteen isolates were MRSA, of which all were *mecA*-positive. Two isolates were resistant to tetracycline, while *tet(k)* was only encoded by one of them and by one phenotypically susceptible isolate; *tet(38)* was encoded by both resistant ones but also by all but one of those susceptible. Out of four that were resistant to rifampicin, only one was found to have genes conferring resistance to it (*rboB_H481Y*, *rboB_L466S*, and *rpoB_S486L*). Resistance to levofloxacin well matched the presence of resistance genes *gyrA_S84L*, *parC_E84G*, *parC_E84K*, *parC_S80F*, and/or *parE_P585S*. While all isolates were susceptible to linezolid, in nine, *23S_C2220T* was found. Out of 23 resistant to erythromycin, 11 encoded *erm(A)*, 4 *erm(C)*, 2 *erm(T)*, and another 2 *msr(A)* and *mph(C)*; in 4, no macrolide resistance genes were detected. For 40 isolates, information on the 30-day survival could be retrieved, as well as for 37 on 90-day survival and 30-day and 90-day outcomes. The only significant difference that was found was that of a lower 30-day survival in patients with *S. aureus* isolates that encoded *splE* (*p* = 0.045; Cramér’s *V* 0.32) but without meeting *χ* ^2^ test assumptions due to low cell frequencies and without significant Fisher exact test (*p* = 0.072). Isolates had an average nucleotide identity to each other of 98.36, ranging from 97.73 to 98.75 (see also **Figure A1 in the Supplementary Material**). The two most represented MLST types were 225 (10) and 22 (9).

#### Streptococcus pneumoniae

In 11 out of 13 cases in which *S. pneumoniae* grew, blood cultures were collected in the EC. The available data were insufficient to perform statistical analyses pertaining to hypotheses involving 30-day and 90-day outcomes and survival rates. Only three isolates had MICs to penicillin sufficiently high to be considered susceptible only at increased dosage (I). Of these, two encoded *pbp1a* and *pbp2b*. Two out of three isolates resistant to erythromycin encoded *erm(B)*. Three out of four isolates resistant to tetracycline encoded *tet(M)*. Isolates had an average nucleotide identity to each other of 98.71, ranging from 98.63 to 98.84. Isolates belonged to 10 different serotypes, the most frequent being 8, with 16F and 17F having two isolates each, and these isolates had 13 different MLST types.

#### Streptococcus pyogenes

In 12 out of 18 cases in which *S. pyogenes* grew, blood cultures were collected in the EC. Here, too, the available data were insufficient to perform statistical analyses pertaining to hypotheses involving 30-day and 90-day outcomes and survival rates. Both isolates resistant to tetracycline carried *tet(M)*. Isolates had an average nucleotide identity to each other of 98.82, ranging from 98.60 to 99.01. The two most represented MLST types were ST28 (5) and ST39 (4).

#### Bacteroides fragilis

The four *B. fragilis* isolates had an average nucleotide identity of 98.97 to each other that ranged from 98.91 to 99.03. One MLST type could not be determined, while the remaining were ST40, ST67, and ST129. The only isolate resistant to clindamycin was also the only one to encode *mef(A)*, as well as the only isolate to have an increased MIC to meropenem was the only one to encode *cfxA*.

#### Escherichia coli

The mean nucleotide identity of *E. coli* isolates ranged from 96.74 to 98.42 (see also **Figure A2 in the Supplementary Material**) with an average of 97.63, with MLST type ST69 being the most frequent (6). Four isolates belonged to ST12, ST73, and ST131 each, and two isolates to ST88 and ST95 each. Two isolates could not be assigned and the remaining isolates all belonged to different MLST types. The most represented O-antigen types were O4, O6, and O25, with four isolates each, and the most represented H-antigen type was H4 (9). The majority of isolates carried one or more plasmids with the most frequent being *IncFIB(APoo1918)* (32), *Col156* (17), *IncFll* (14), and *IncFlA* (13). The significant differences found were only that of a lower 30-day and 90-day survival in patients with isolates encoding *IncFll(pSE11)* and *p0111* (*p* = 0.001, Cramér’s *V* = 0.69) but without *χ* ^2^ test assumptions being met due to low cell frequencies.

#### Klebsiella pneumoniae

For three *K. pneumoniae* isolates, the MLST type could not be determined, and another three belonged to ST147. Six MLST types were represented twice (ST14, ST15, ST20, ST78, ST661, and ST3328), and another 22 just once. With Kleborate, the missing MLSTs were identified as ST307, ST223-1LV, and ST1013-1LV. Isolates had an average nucleotide identity of 99.03 to each other, ranging from 98.93 to 99.12 (see also **Figure A3 in the Supplementary Material**), and carried an average of 2.4 plasmids, ranging from two isolates carrying none to three carrying five. The number of encoded stress tolerance, AMR, and virulence genes varied from none to 36, from none to 27, and from none to 12, respectively, with only one isolate encoding neither of each. significant differences found were that of a lower 30-day and 90-day survival in patients with isolates encoding *iucA*, *iucB*, and *iucC* (*p* = 0.039, Cramér’s *V* = 0.35) and *iroB* and *iroN* (*p* = 0.039, Cramér’s *V* = 0.35), but all violating *χ* ^2^ test assumptions due to low cell frequencies, and for *aac(3)-IId*, *aph(3′)-VI*, *gyrA_D87G*, and *phoQ_R16C* (*p* = 0.046, Cramér’s *V* = 0.34) and *blaSHV*, *dfrA1*, *iucC*, and *ompK36_D135DGD* (*p* = 0.039, Cramér’s *V* = 0.35), additionally without significant Fisher exact test. The four isolates encoding *blaOXA-48* and the three isolates encoding *blaNDM-1* were all correctly identified as such in routine diagnostics.

#### *Enterobacter* spp.

The sequenced *Enterobacter* isolates were all reported in routine diagnostics as *Enterobacter cloacae* complex isolates, but WGS revealed these to be *E. quasihormaechei* (31), *E. quasiroggenkampii* (4), *E. sichuanensis* (3), *E. cloacae* (2), *E. chengduensis* (2), and *E. wuhouensis* (1). Isolates either encoded no virulence genes or three (*iroB*, *iroC*, *iroN*), with all isolates encoding three being *E. quasihormaechei* isolates. The number of carried AMR and stress response genes varied significantly among the isolates, ranging from 1 to 15 for AMR genes and 1 to 35 for stress response genes. Isolates encoded between none and four plasmids, the most frequent being *IncFII(pECLA)* (12), *IncFIB(pECLA)* (10), and *Col440I* (8). The one *blaVIM-1*-positive isolate was phenotypically susceptible to imipenem and meropenem and was, hence, not investigated for carrying carbapenemases in routine diagnostics. MLST type could not be determined in 11 cases; in seven, ST50 was identified, and in three, ST118 was identified. Other MLST types were only represented once or twice. No significant differences were found regarding the 30-day and 90-day survival and the presence or absence of investigated genes.

#### *Citrobacter* spp.

Four *Citrobacter* isolates were *C. freundii*, another four were *C. braakii*, and seven were *C. koseri*. The average nucleotide identity of *C. koseri* (98.88) and *C. freundii* (98.59) isolates was higher than that of *C. braakii* (94.41) isolates, despite *C. freundii* isolates belonging to four different MLST types. Among all eight *C. freundii* and *C. braakii* isolates, there was only one isolate carrying virulence genes (*ybtP* and *ybtQ*), whereas all *C. koseri* isolates encoded the same virulence factors (*iucA*, *iucB*, *iucC*, *ybtP*, and *ybtQ*) with the exception of *senB*, which was only encoded by four. Isolates encoded between one and three AMR genes. The most significant variation was observed in the number of stress response genes encoded by the isolates. Two *C. braakii* isolates and six *C. koseri* isolates only encoded a single stress response gene, which was *fief*, while the remaining *C. koseri* isolates encoded six, the remaining *C. braakii* isolates encoded 17, and *C. freundii* isolates encoded between 24 and 35. Isolates carried from none to four plasmids with not a single plasmid carried by more than one isolate. The only two *Citrobacter* isolates encoding *qnrB38* were the only ones to have MICs to moxifloxacin above the lowest measurable level.

#### Proteus mirabilis

*Proteus mirabilis* isolates had an average nucleotide identity of 99.12 to each other, ranging from 98.83 to 99.25. The significant differences found were that of a lower 30-day survival when isolates encoded *aph(3′)-la* (*p* = 0.028; Cramér’s *V* 0.63); however, *χ* ^2^ test assumptions were violated due to low cell frequencies and the Fisher exact test was not significant (*p* = 0.091). All isolates carrying *blaTEM-1* were resistant to ampicillin and all but one isolate to ampicillin/sulbactam. All isolates resistant to trimethoprim/sulfamethoxazole carried *dfrA1* and in addition either *sul1* or *sul2*. Four isolates carried an *IncQ1* plasmid.

#### Serratia marcescens

More than half of the isolates were isolated from patients in the ICU, but isolates only had an average nucleotide identity to each other of 95.79, ranging from 95.18 to 96.00 (see also **Figure A4 in the Supplementary Material**). No significant differences were found in the 30-day and 90-day survival linked to the presence or absence of certain genes, age, or ward type. Four isolates carried a *pSM22* plasmid and another three isolates either *Col440I*, *IncX5*, or *IncX6*. The *IncX5* carrying isolate carried a *blaVIM-1* alongside it that was neither specifically investigated nor detected in routine diagnostics. That isolate was resistant to piperacillin–tazobactam, tested cephalosporins, and imipenem but had a MIC to meropenem of 0.19 as determined by the gradient strip test (Liofilchem, Roseto degli Abruzzi, Italy). It was also the only one to be *sul1*-positive although it was just as susceptible to trimethoprim/sulfamethoxazole as all the other isolates. Isolates carried four or five out of 10 stress response genes and either five or six out of 13 AMR genes. The most frequent AMR genes were *ssmE*, *smdB*, *smdA*, *sdeY*, *sdeA* (27 each), *smfY*, *sdeB* (26 each), *aac(6′)* (25), *blaSRT* (22), and *tet(41)* (21).

#### *Acinetobacter baumannii* complex

Among the isolates of the *A. baumannii* complex, four were *A. baumannii* isolates and eight were *A. pittii*. Among the *A. baumannii* isolates, the MLST types represented were ST1, ST40, and ST213, with one isolate unassigned and with an average nucleotide identity to each other ranging from 97.54 to 97.69 and from 96.52 to 98.03 among the *A. pittii* isolates. No virulence genes were identified; however, between one and five stress tolerance genes were identified in all isolates and either five or six AMR genes in *A. pittii* and between 3 and 12 in *A. baumannii*. In both species, the most frequently encoded stress tolerance genes were *nreB* (12), *clpK* (6), *yfdX2* (5), *trxLHR* (5), and *hdeD-GI* (5), while the most frequent AMR genes were *amvA* (12) and *ant(3″)-IIa* (10). The available data were insufficient to perform statistical analyses pertaining to hypotheses involving 30-day and 90-day outcomes and survival rates.

#### Pseudomonas aeruginosa

The average nucleotide identity of *P. aeruginosa* isolates was 98.85 and ranged from 98.66 to 98.94. For 11 isolates, no MLST type could be determined, while three belonged to ST234, ST253, and ST823 each, two to ST308, ST316, and ST446 each, and all the remaining isolates to different MLST types. Isolates belonged mainly to serogroups O11 (13), O6 (9), O1 (5), and O10 (4). No virulence genes were detected, and stress response genes were only detected in less than half of the isolates in which they ranged from 2 to 14 in number. The most frequently encoded were *merE* (14), *merT* (12), *merR* (12), *merP* (12), *merD* (11), and *merA* (11). No isolate had fewer than 8 AMR genes with some carrying up to 18. By far, the most frequent were *mexE* (38), *mexA* (38), *fosA* (38), *aph(3′)-llb* (38), *mexX* (37), *catB7* (37), and *crpP* (29). Thirty-five isolates carried *blaOXA* genes and four *blaVIM* (three *blaVIM-2* and one *blaVIM-11*). Five isolates were classified as 4MRGN and three as 3MRGN. One *blaVIM-2* encoding isolate did not previously classify as 4MRGN as it had below-resistant MICs to piperacillin–tazobactam and cefepime and the *blaVIM-2* gene was neither investigated nor detected in routine diagnostics. It was resistant to imipenem, meropenem, ciprofloxacin, and ceftazidime. No significant differences were found in the 30-day and 90-day survival linked to the presence or absence of certain genes. Isolates exhibited a distinct clustering pattern, roughly partitioning into three distinct branches. Notably, one of these branches encompassed all but one isolate from patients with negative 30-day survival (see **Figure A5 in the Supplementary Material**) (*p* = 0.038).

#### Stenotrophomonas maltophilia

*Stenotrophomonas maltophilia* isolates only had an average nucleotide identity of 93.15 to each other, ranging from 92.09 to 94.17. One MLST type could not be determined, while all remaining were of different ones: ST4, ST23, ST224, and ST233. Only one did not have any stress response genes, the others had from three to nine, and all isolates carried either seven or eight antimicrobial resistance genes. Those shared among all isolates were *aph(6)*, *emrA*, *emrB*, *emrC*, and *smeF*.

## Discussion

The present study aimed to conduct a comprehensive analysis of blood culture isolates’ genomes, with a retrospective correlation between the detected virulence genes, resistance genes, and stress tolerance genes with the phenotypical susceptibilities and patient outcomes. The findings of this investigation yielded extensive and informative data on the distribution of genes implicated in bloodstream infections across various species. However, the interpretation of these results necessitates careful consideration due to several noteworthy factors. The intriguing observed associations between survival outcomes and the presence of specific resistance genes warrant caution, particularly given the limited knowledge regarding many of the identified virulence factors. The *χ* ^2^ test assumptions were persistently violated due to the relatively small sample sizes in the groups of patients with negative 30-day and 90-day survival available for each species, making it imperative to validate and strengthen these associations by replicating the study with larger sample sizes for different species. One key consideration is the possibility that host factors may play a more significant role than pathogen factors in determining the outcomes of bloodstream infections and the inherent difficulty in fully accounting for such factors in studies of this nature (Hekker et al., 2000; Casadevall and Pirofski, 2001; Tseng et al., 2002; Beck et al., 2004; Tseng et al., 2005; Newman et al., 2017).

For example, research on *splE* in *S. aureus* still aims to uncover its precise functions and mechanisms. It seems that *splE* plays a significant role in *S. aureus* pathogenesis by promoting immune evasion through the degradation of immune components and facilitating tissue invasion by breaking down extracellular matrix proteins (Stach et al., 2018). Confirming its role in survival would further emphasize the importance of understanding *splE* for developing effective strategies against staphylococcal infections. The *IncFll(pSE11)* and *p0111* plasmids that were linked to lower survival in *E. coli* are known for disseminating AMR genes (Tasleem Jan and Tiwari, 2017; Wang et al., 2022), and their likely correlation with factors such as prolonged hospitalizations cannot be excluded to confound results, emphasizing that interpretation of the results warrants considered caution. The lower survival in *aph(3′)-la* encoding *P. mirabilis* isolate is probably the most questionable finding, because aside from resistance to aminoglycosides, it is not reported to contribute to virulence (Shaw et al., 1993), and aminoglycosides are infrequently used in our setting.

The study highlights important differences in genetic variation across different species and might be representative to some degree for these pathogens involved in BSI, while it is certainly important to exercise caution in generalizing these findings or applying them to other settings. The identity and coverage values for *E. faecium* ST117 and *E. coli* ST131 were both 100% for their exact alleles. Notably, ECO 131 has been highlighted in several studies as a rapidly expanding multidrug-resistant pathogen with high receptivity and the potential to develop resistance to last-resort antibiotics such as carbapenems and colistin (Pitout and Finn, 2020; Li et al., 2021; Taati Moghadam et al., 2021; Brumwell et al., 2023). Serotypes H4 and O25 were found to match all isolates in our investigation, consistent with previous studies (Brumwell et al., 2023), but none of the isolates exhibited the antimicrobial MDR profile of 3MRGN or 4MRGN. *Enterococcus faecium* ST117 was previously identified as a rising factor in vancomycin-resistant enterococci (VRE) (Weber et al., 2020), and five out of six isolates in our study were found to carry *vanB* genes. A comparison with this recent comprehensive study involving 120 isolates revealed that all the genes mentioned in the study (*msrC*, *efmA*, *erm(B)*, *dfrG*, *aac(6′)-Ii*, *gyrA*, *parC*, and *pbp5*) matched those present in our isolates, except for *efmA*, which was absent in the NCBI database used. The notable clustering of ST50 *E. quasihormaechei* isolates calls for focused investigations, given the escalating local and global issue of sewage-related clonal colonization in hospital sanitary facilities (Babouee Flury et al., 2016; Kehl et al., 2022; Stokes et al., 2022). Similarly, the recurring identification of ST147 *K. pneumoniae* and ST235 and ST823 *P. aeruginosa* isolates, even in wards geographically distant from those assumed to have a contamination source in our setting (Kehl et al., 2022; Neidhöfer et al., 2023a), urges us to implement transmission dynamics monitoring networks (Bohl et al., 2022; Ko et al., 2022; Neidhöfer et al., 2023b).

## Conclusion

Conducting replication studies with larger sample sizes for each species is imperative to cautiously interpret associations between survival outcomes and the presence of specific genes, emphasizing the need for further research in this area. A deeper understanding of virulence and host factors will not only aid in the interpretation of results but also pave the way for the development of more targeted therapeutic and preventive measures, thus enhancing patient outcomes. In addition, implementing more efficient transmission dynamics monitoring holds significant promise as a valuable tool in preventing bloodstream infections caused by certain pathogens, highlighting the importance of establishing robust surveillance systems.

## Data availability statement

All data and original contributions presented in the study are included in the article/**Supplementary Material**, except for genomes that cannot be readily made available because of binding collaboration agreements. These can, however, be made available upon request directed to the corresponding author.

## Ethics statement

The studies involving humans were approved by the Ethics Committee of the University Hospital Bonn. The studies were conducted in accordance with the local legislation and institutional requirements. Written informed consent for participation was not required from the participants or the participants’ legal guardians/next of kin in accordance with the national legislation and institutional requirements.

## Author contributions

CN: Conceptualization, Data curation, Formal Analysis, Investigation, Methodology, Project administration, Supervision, Visualization, Writing – original draft, Writing – review & editing. MN: Formal Analysis, Investigation, Methodology, Software, Visualization, Writing – original draft, Writing – review & editing, Conceptualization, Data curation. RJ: Data curation, Formal Analysis, Methodology, Project administration, Writing – review & editing. BA: Data curation, Writing – review & editing. SU: Data curation, Methodology, Software, Writing – original draft, Writing – review & editing. UN: Data curation, Methodology, Software, Writing – original draft, Writing – review & editing. SG: Data curation, Methodology, Software, Writing – original draft, Writing – review & editing. MP: Conceptualization, Funding acquisition, Project administration, Resources, Supervision, Writing – review & editing.
