# Supplementary material for: Exploring clonality and virulence gene associations in bloodstream infections using whole-genome sequencing and clinical data
Source: Front Cell Infect Microbiol. 2023 Nov 14;13:1274573. doi: 10.3389/fcimb.2023.1274573 (PMC10682671; doi:10.3389/fcimb.2023.1274573)
Supplement: Supplementary file 1 [file DataSheet_1.pdf]

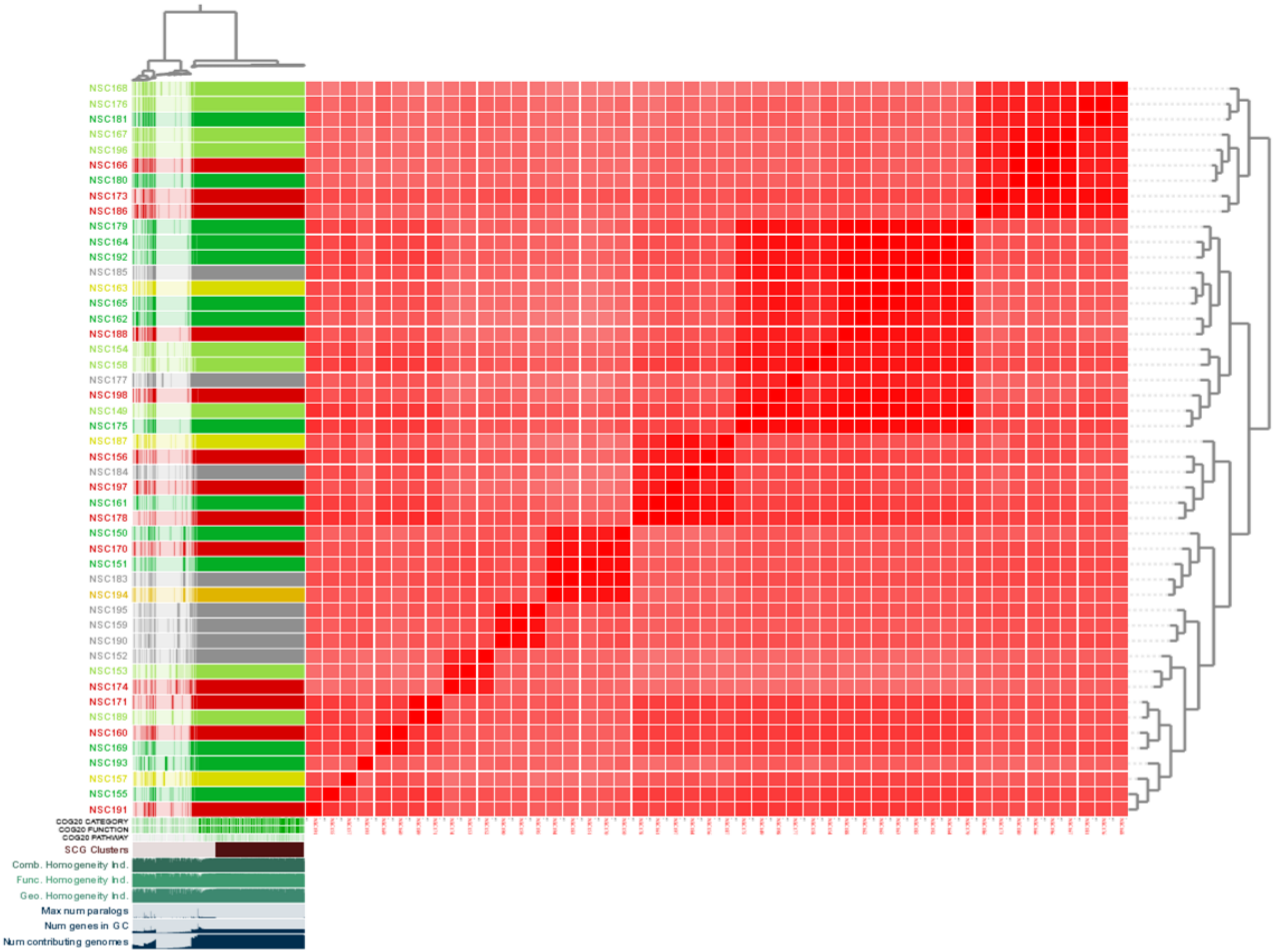

Figure A1: Phylogenetic tree and ANI heatmap of *S. aureus* isolates. Isolates are colored depending on patient outcome. Green: 30 day and 90 day survival positive and patient discharged after 30 days; Light Green: 30 day and 90 day survival positive and patient still hospitalized after 30 days; Yellow: 30 day survival positive and patient still hospitalized, no information about 90 day survival; Orange: 30 day survival positive but 90 day survival negative; Red: 30 day survival negative; Gray: Insufficient information avaiable. The saturation of the squares corresponds to the respective match from low: comparatively low genomic match to high: 100% genomic match.

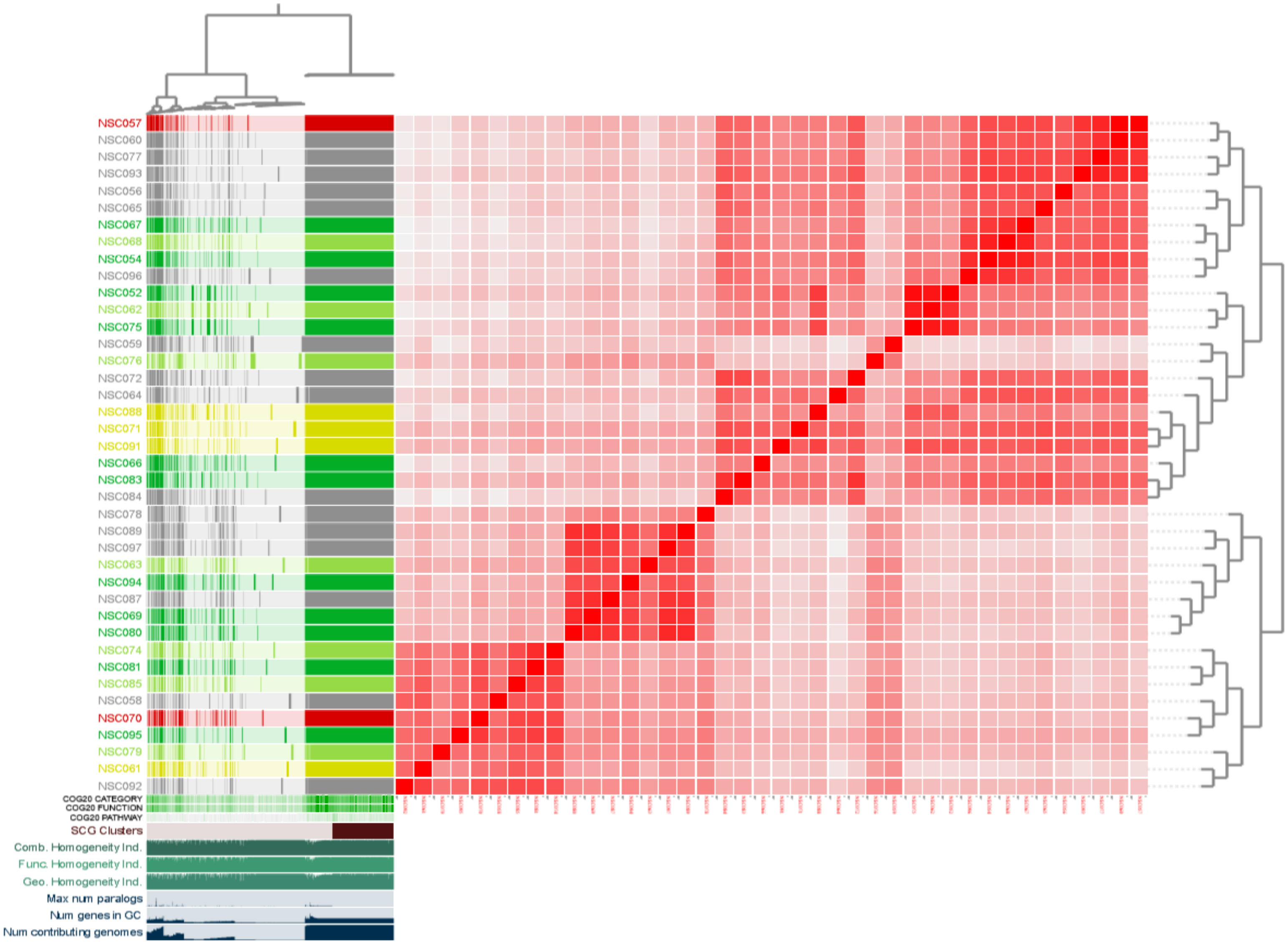

Figure A2: Phylogenetic tree and ANI heatmap of *E.coli* isolates. Isolates are colored depending on patient outcome. Green: 30 day and 90 day survival positive and patient discharged after 30 days; Light Green: 30 day and 90 day survival positive and patient still hospitalized after 30 days; Yellow: 30 day survival positive and patient still hospitalized, no information about 90 day survival; Orange: 30 day survival positive but 90 day survival negative; Red: 30 day survival negative; Gray: Insufficient information avaiable. The saturation of the squares corresponds to the respective match from low: comparatively low genomic match to high: 100% genomic match.

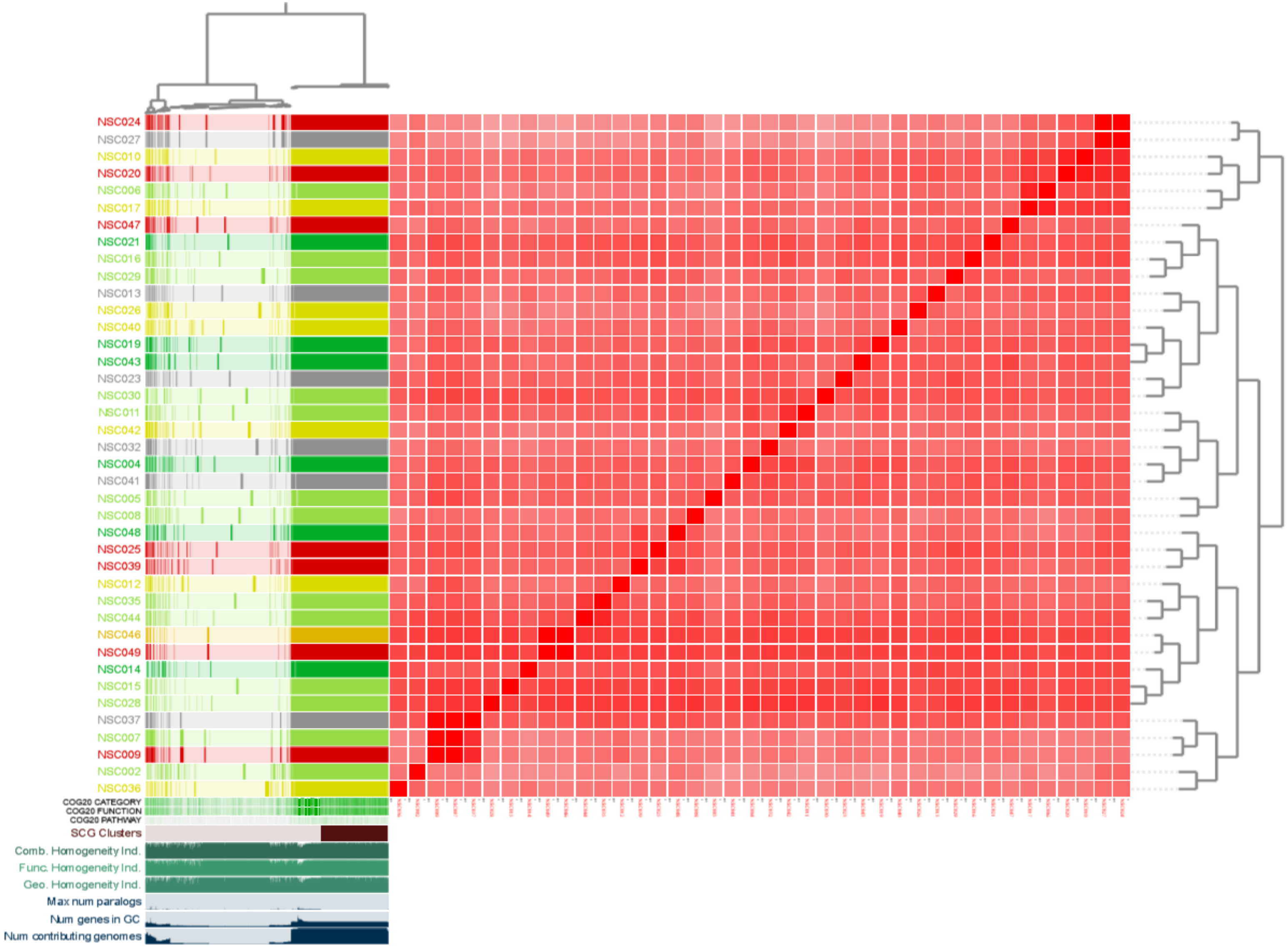

Figure A3: Phylogenetic tree and ANI heatmap of *K. pneumoniae* isolates. Isolates are colored depending on patient outcome. Green: 30 day and 90 day survival positive and patient discharged after 30 days; Light Green: 30 day and 90 day survival positive and patient still hospitalized after 30 days; Yellow: 30 day survival positive and patient still hospitalized, no information about 90 day survival; Orange: 30 day survival positive but 90 day survival negative; Red: 30 day survival negative; Gray: Insufficient information avaiable. The saturation of the squares corresponds to the respective match from low: comparatively low genomic match to high: 100% genomic match.

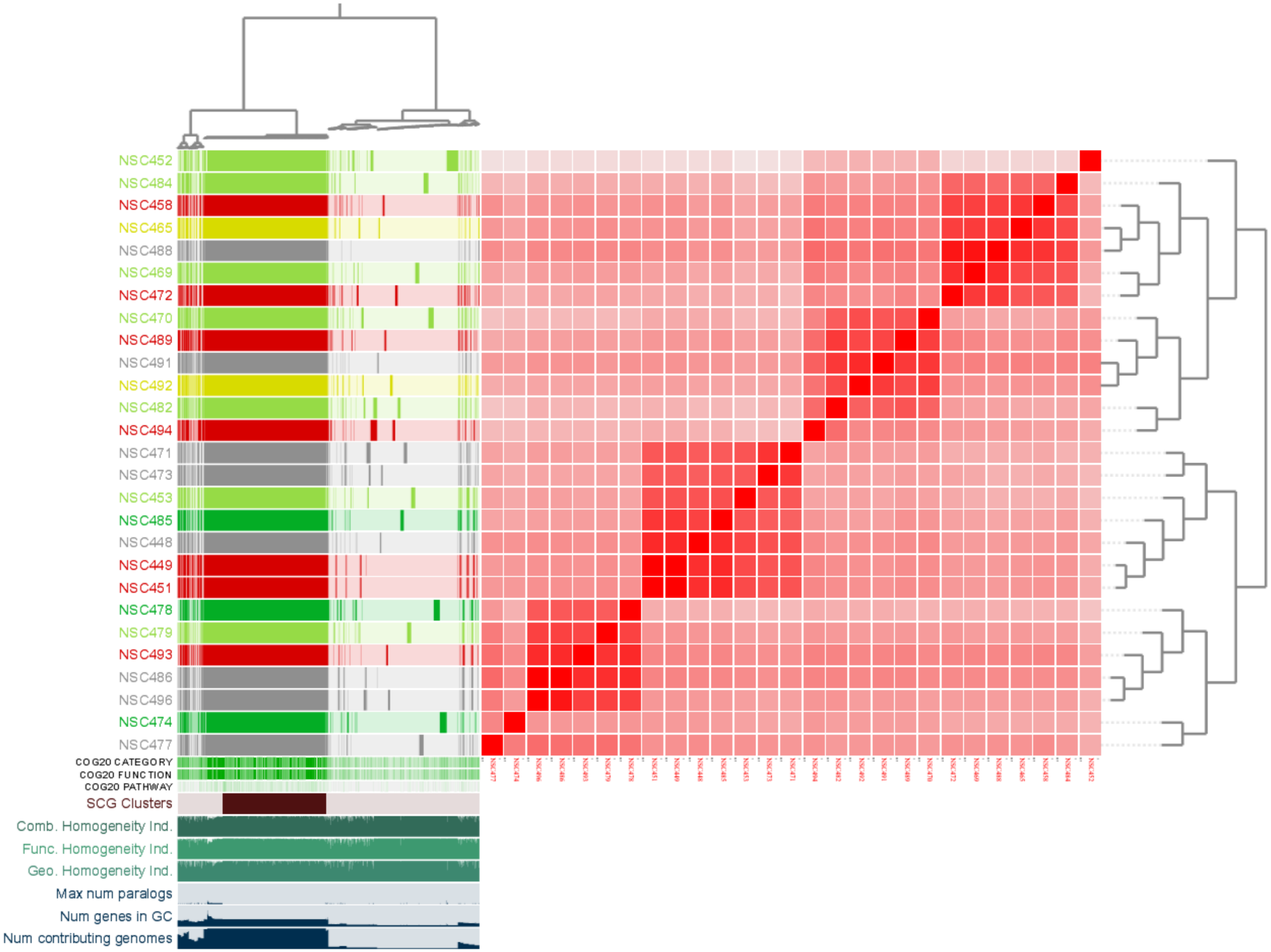

Figure A4: Phylogenetic tree and ANI heatmap of *S. marcescens* isolates. Isolates are colored depending on patient outcome. Green: 30 day and 90 day survival positive and patient discharged after 30 days; Light Green: 30 day and 90 day survival positive and patient still hospitalized after 30 days; Yellow: 30 day survival positive and patient still hospitalized, no information about 90 day survival; Orange: 30 day survival positive but 90 day survival negative; Red: 30 day survival negative; Gray: Insufficient information avaiable. The saturation of the squares corresponds to the respective match from low: comparatively low genomic match to high: 100% genomic match.

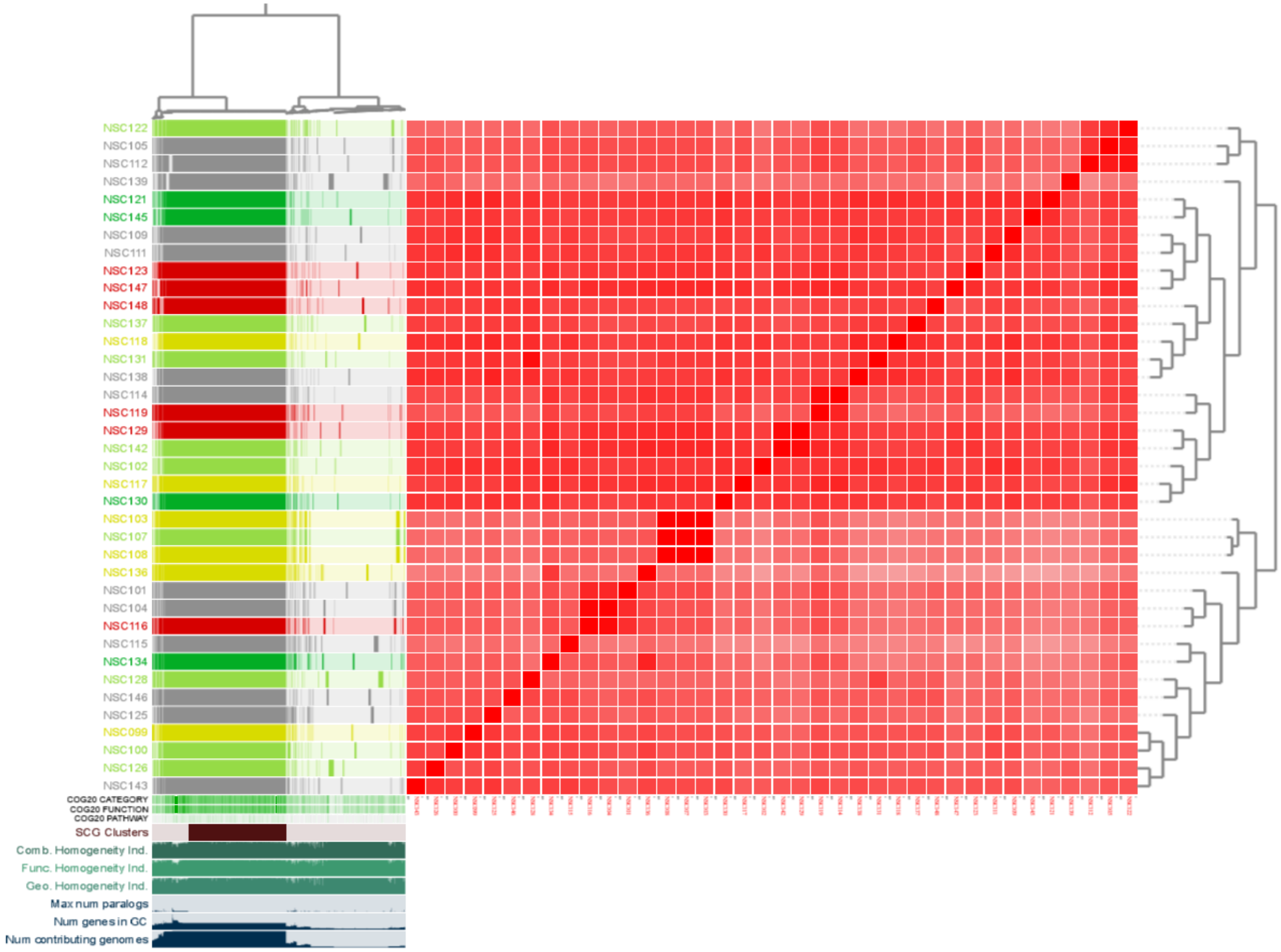

Figure A5: Phylogenetic tree and ANI heatmap of *P. aeruginosa* isolates. Isolates are colored depending on patient outcome. Green: 30 day and 90 day survival positive and patient discharged after 30 days; Light Green: 30 day and 90 day survival positive and patient still hospitalized after 30 days; Yellow: 30 day survival positive and patient still hospitalized, no information about 90 day survival; Orange: 30 day survival positive but 90 day survival negative; Red: 30 day survival negative; Gray: Insufficient information avaiable. The saturation of the squares corresponds to the respective match from low: comparatively low genomic match to high: 100% genomic match.
